# Metabolic robustness is an emergent property of hierarchical network organization in yeast

**DOI:** 10.1128/msystems.00276-26

**Published:** 2026-05-18

**Authors:** Mohammad Tauqeer Alam

**Affiliations:** 1 Department of Biology, College of Sciences, United Arab Emirates University 105960, Al Ain, Abu Dhabi, United Arab Emirates; Korea Advanced Institute of Science and Technology, Daejeon, Republic of Korea

**Keywords:** metabolic network, robustness, systems biology, robust metabolite, flux reprogramming, adaptation

## Abstract

Biological systems exhibit intrinsic robustness, allowing cells to sustain growth despite diverse perturbations. We quantified the inherent robustness of the *Saccharomyces cerevisiae* genome-scale metabolic network by globally perturbing metabolite production fluxes using a hypothetical sink reaction. Among the 317 high-flux active metabolites, excluding macromolecular intermediates and highly connected cofactors, 85% were found to be robust. Of these robust metabolites, more than half (144/269) were overproduced under perturbation compared with minimal-media controls. These metabolites, mapped to a single central metabolic cluster within the metabolic network, were enriched in core biosynthetic pathways and were largely growth-essential, indicating that the network tolerates elevated biosynthetic demand for most key metabolites. Flux- and pathway-level analyses revealed a coordinated adaptive program involving activation of alternative routes at the network periphery and extensive flux redistribution within central metabolism. Central carbon metabolism and oxidative phosphorylation were broadly suppressed, whereas the pentose phosphate, shikimate, and lipid-related pathways were selectively reinforced to support NADPH generation and redox balance. This reorganization establishes an energy-efficient, redox-stabilized metabolic state that underlies system-wide resilience. Together, these findings show that metabolic robustness emerges from a hierarchical network architecture coupling a stable core with flexible peripheral adaptation. This framework explains cellular resilience and offers design principles for engineering robust, damage-resistant metabolic systems.

**IMPORTANCE:**

In this study, we investigated the intrinsic robustness of the metabolic network and uncovered a structured organization characterized by a conserved central core and a flexible, peripherally rewired subsystem. Our results suggest that this architecture reflects an evolutionary balance between stability and adaptability. By systematically perturbing more than 300 metabolites, we provide comprehensive and consistent evidence supporting the existence of this core–periphery organization. These findings advance our understanding of how metabolic systems maintain functional stability while retaining the capacity for adaptive rewiring.

## INTRODUCTION

Living cells are continually exposed to unpredictable environmental and genetic perturbations that often impose challenges on cellular homeostasis and metabolic balance (1–4). To function effectively under such fluctuating conditions, cellular systems have evolved robustness, a fundamental property of every biological system (5–11). For instance, many genes can be deleted without causing lethality due to compensatory mechanisms embedded within genetic interaction networks (12–15). Similarly, organisms often exhibit consistent phenotypes despite underlying genetic or environmental variation during development (6). Furthermore, cellular signaling pathways, such as the MAPK cascade in yeast and mammalian cells, maintain stable and reproducible outputs despite fluctuations in upstream signals (16, 17).

The metabolic network, the most extensive and interconnected system within the cell, has likewise evolved a high degree of robustness to maintain functionality under diverse perturbations (7, 9, 11, 18–20). Cells often adopt metabolic strategies that prioritize energy conservation and redox homeostasis over maximal biomass yield. Metabolic robustness enables cells to reprogram flux distributions, activate alternative pathways, and engage intracellular regulatory mechanisms to sustain growth and essential metabolic functions under environmental or genetic stress (11, 18, 21–29).

Despite the fundamental importance of metabolic systems and the inherent unpredictability of environmental conditions, our understanding of metabolic network robustness under complex metabolic demands remains limited. The extent to which a metabolic network can withstand production stress—and for which metabolites—remains unclear. In particular, the mechanisms that enable cells to maintain optimal growth under conditions of metabolic overproduction are not yet fully understood.

In this study, we systematically investigate the mechanisms underlying metabolic robustness by introducing a range of metabolic perturbations. Using the Yeast9 genome-scale metabolic model (30), we identify robust metabolites under metabolite overproduction demands. We further assess the extent to which cells reorganize their metabolism to accommodate these additional demands and explore how network hierarchy and topological organization contribute to metabolic robustness.

## MATERIALS AND METHODS

### Characterization of robust metabolites in *S. cerevisiae*

Cellular metabolites are considered robust if perturbations in their production levels do not significantly impact cellular growth. To systematically identify such robust intracellular metabolites, we utilized the Yeast9 genome-scale metabolic model (30) of *Saccharomyces cerevisiae*. First, we simulated the model using the flux balance analysis (FBA) framework to predict the maximum growth rate under defined minimal media constraints (31). The resulting optimal growth rate and associated flux distribution were treated as the control condition. For each metabolite in the model, we then computed its total production and consumption rates by summing the fluxes of all reactions contributing to its synthesis and utilization, respectively. Because FBA assumes a steady-state condition (31, 32), the production and consumption rates are equal. We then identified metabolites that were active under the control condition, defined as those exhibiting an overall production flux greater than 1 × 10⁻⁴ mmol/gDW/h. From this set (740 metabolites in total), we excluded macromolecular intermediates (e.g., precursors of biomass components such as RNA, DNA, and proteins), highly connected cofactors (e.g., ATP, NADH, NADPH, and CoA), as these participate in numerous reactions and are not informative for robustness characterization, and metabolites with low overall production flux (< 0.001 mmol/gDW/h).

For the remaining metabolites (317 in total, File S1), we assessed their robustness by introducing a perturbation to their overall production while maintaining the same growth and environmental conditions as the control. Specifically, for each metabolite, we added a hypothetical sink reaction representing an artificial “waste” or diversion pathway. The flux through this sink reaction was constrained to 10% of the metabolite’s total consumption flux under the control condition, simulating an additional, unexpected metabolic demand. We then fixed the model’s growth rate and nutrient uptake rates to those of the control condition and re-optimized the model to predict the new flux distribution. If the model could sustain at least 10% of the control growth rate under this perturbation, the metabolite was classified as robust (269 in total). Conversely, if the growth rate dropped more than 90% of the control level, the metabolite was considered non-robust (48 in total), indicating that its overproduction demand imposed a significant metabolic burden that the cell could not fully compensate for. For each robust metabolite, we increased the cellular demand by setting the sink reaction flux to 10%, 20%, …, and up to 100% of its total production flux. The growth rate and nutrient uptake rates were fixed to match those under the control minimal medium condition.

For each metabolite classified as robust, we further quantified the metabolic adjustment by calculating the ratio of its total production rate under the perturbed condition to that under the control condition. This ratio reflects how the metabolic network compensates for the imposed additional demand (the hypothetical sink reaction). If the production ratio was less than 1, the metabolite was considered underproduced (125 out of 269 robust metabolites), indicating that the cell was able to achieve maximum growth with reduced levels. Conversely, if the production ratio was greater than 1, the metabolite was categorized as overproduced. This outcome (observed for 144 out of 269 robust metabolites) suggests that the metabolic network was able to compensate for the additional sink demand and, in fact, has increased the overall production rate. Finally, we analyzed the flux distributions under the overproduction demand of these metabolites to investigate the underlying mechanisms of robustness in the metabolic network of *Saccharomyces cerevisiae*.

### Model simulation and metabolite essentiality

To examine the systemic response of the metabolic network to metabolite overproduction, the genome-scale metabolic model was simulated using the flux balance analysis (FBA) framework implemented in the COBRA Toolbox (31). For each overproduction condition corresponding to a robust metabolite, reaction fluxes and total metabolite production fluxes were compared with those obtained under the control condition. Metabolites and reactions were classified into four categories based on their relative flux changes: activated, deactivated, increased flux, or decreased flux.

A metabolite was defined as activated if its total production rate was zero (<1e-4) in the control but nonzero (>1e-4) under the overproduction condition, and deactivated if the opposite occurred. Metabolites exhibiting a total production flux at least 10% higher than in the control were categorized as increased flux, while those with a 10% lower production flux were classified as decreased flux. The same definition was applied for reaction fluxes. Exchange fluxes were analyzed, and the frequency of metabolite export across all overproduction conditions was computed.

Metabolite essentiality was assessed by constraining all reactions involving a given metabolite to zero flux and simulating growth under minimal media conditions using FBA. If the model failed to produce biomass under the metabolite-deleted condition, the metabolite was classified as essential; otherwise, it was considered non-essential.

### Network topological analysis

Network topological analysis and visualization were conducted using the igraph package in R (33). The metabolic network was derived from the original Yeast9 model (30) and reduced based on active reactions (flux >1 × 10⁻⁴) for subsequent analysis. Reactions exhibiting nonzero flux under any simulated condition were retained for network construction. Macromolecular intermediates (e.g., DNA, RNA, proteins, lipids, and carbohydrates), common cofactors (e.g., H^+^, H₂O, ATP, ADP, AMP, Pi, coenzyme A, CO₂, NADH, and NAD^+^), extracellular metabolites, inorganic ions, self-connections, duplicate reactions, and highly connected hub metabolites (degree >50) were excluded. Finally, small disconnected subnetworks containing two or fewer metabolites were removed, resulting in a refined active metabolic network used for topological characterization.

Within the metabolic network, the shortest path length between each robust metabolite and all other metabolites was calculated using the *shortest.paths* function in the igraph package in R (33). This analysis quantified the topological proximity of robust metabolites to other nodes in the network. Network peripheral and central nodes were identified based on eccentricity and betweenness centrality measures, respectively (33). Metabolites with eccentricity values greater than the network’s average eccentricity plus one standard deviation were classified as peripheral. For identifying central metabolites, betweenness centrality values were log₂-transformed, and metabolites with values exceeding the mean plus one standard deviation were designated as central nodes within the network. We have the igraph package in R for these network features (33).

### Pathway enrichment

Enrichment analysis of different reaction categories such as activated, deactivated, increased flux, and decreased flux, within the metabolic pathways of the Yeast9 model (30), was performed using a hypergeometric test. For example, the statistical significance of the enrichment of activated reactions in a given pathway was computed using the *phyper(x−1, m, n, k, lower.tail = FALSE*) function in R, where *m* represents the total number of activated reactions, *n* the number of remaining reactions, *k* the total number of reactions in the pathway, and *x* the number of activated reactions observed within that pathway.

## RESULTS

### Identification of robust metabolites and adaptive metabolic responses

**Fig 1:**
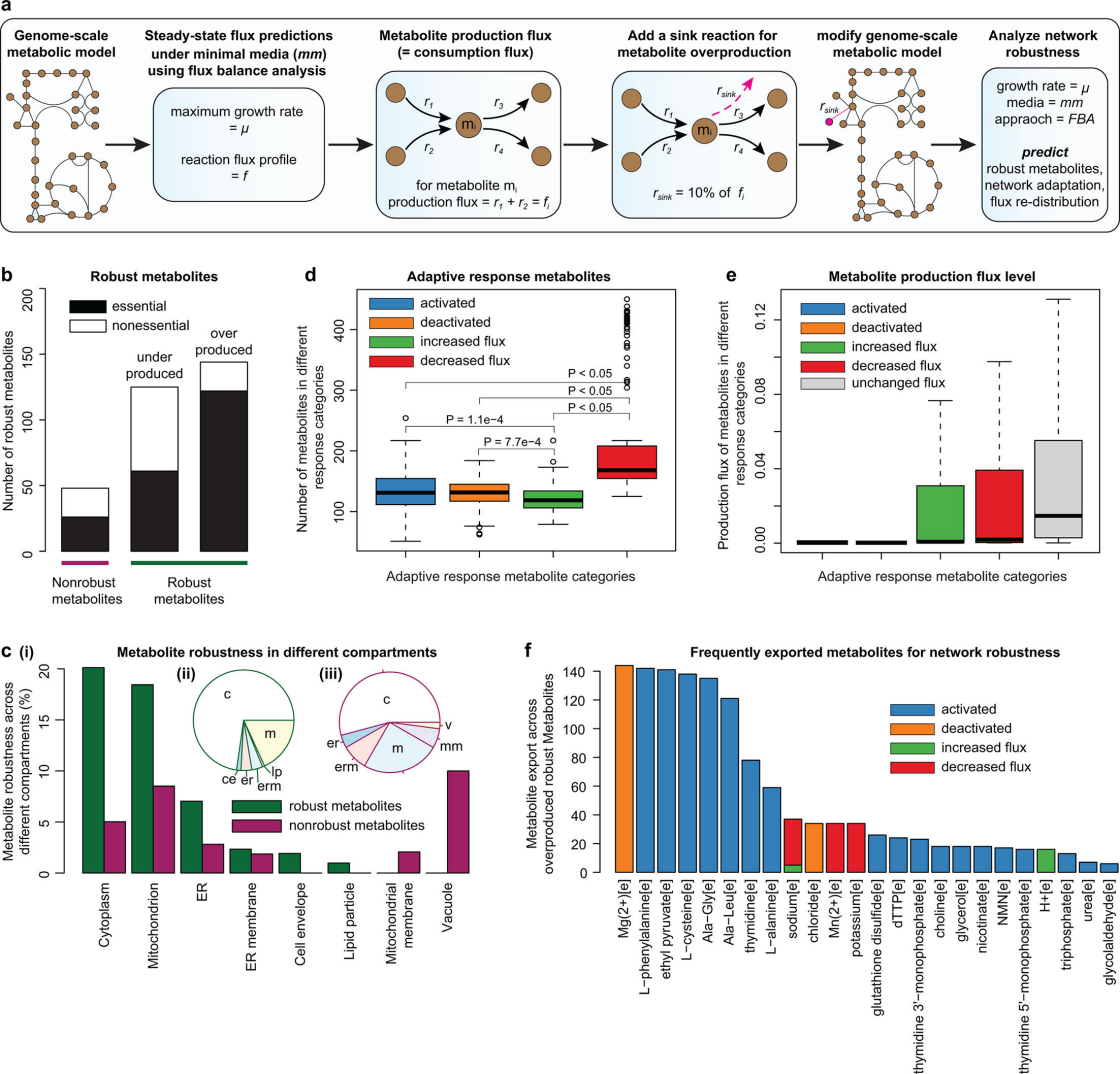
Identification of robust metabolites and associated overflow metabolism. (**a**) Schematic illustrating metabolite overproduction demand through the introduction of a hypothetical sink reaction into a genome-scale metabolic model for the identification and characterization of robust metabolites. (**b**) Number of robust and non-robust metabolites within the metabolic network, highlighting the percentage of essential metabolites within each group. (**c**) Proportion of overproduced robust and non-robust metabolites across cellular compartments. (**d**) For each overproduced robust metabolite, the number of affected metabolites and (**e**) total production flux levels for cellular metabolites in different categories, including activated, deactivated, and those with increased or decreased production fluxes, are presented. (**f**) For each overproduced robust metabolite, the frequency of different exported metabolites is presented.

Cellular metabolites are considered robust when the metabolic network can adjust and reroute fluxes through alternative pathways without affecting cellular growth. To identify robust metabolites, we systematically analyzed changes in metabolite production across the metabolic network under identical nutrient uptake and growth rate conditions (30, 31). Among the 2,805 metabolites represented in the Yeast9 model (30) across various cellular compartments, 740 were produced under minimal medium conditions that supported maximal growth (production flux >1 × 10⁻⁴ mmol/gDW/h). Highly connected cofactors, macromolecular intermediates, and metabolites with low overall production flux (< 0.001 mmol/gDW/h) were excluded from further analysis to focus on high-flux metabolic intermediates with more independent flux control. In total, the robustness of 317 metabolites (File S1) was evaluated by perturbing their production capacity with the same growth as unperturbed control (Fig. 1a). For each metabolite, the total production rate was first quantified by summing the fluxes of all reactions contributing to its synthesis in the control (unperturbed) minimal medium condition (see *Methods* for details) (Fig. 1a). Next, a hypothetical sink reaction was introduced for each metabolite, constraining its flux to a minimum of 10% of the control production rate, effectively simulating a “waste” pathway that diverted part of its flux (Fig. 1a). The model was then re-optimized under the same nutrient uptake constraints to maintain constant biomass yield while accommodating the additional production demand, allowing us to assess how the network reorganizes to preserve metabolic functionality (Fig. 1a).

Of the 317 metabolites analyzed, 269 were classified as robust, indicating that the metabolic network could accommodate altered production levels without compromising biomass synthesis (Fig. 1b). Within this group, 125 metabolites exhibited reduced production under perturbation, suggesting compensatory flux redistribution, while 144 metabolites maintained or increased their production to meet the elevated demand. In contrast, 48 metabolites were identified as non-robust, as perturbing their synthesis disrupted network balance and prevented the model from sustaining biomass production under the imposed conditions (Fig. 1b; File S1). Notably, 85% of the overproduced robust metabolites (122 out of 144) were essential, whereas only about half of the non-robust (54%; 26/48) and downregulated robust metabolites (49%; 61/125) were essential for growth (Fig. 1b). This high percentage of essentiality among robust metabolites indicates that robustness is embedded within the metabolic core supporting fundamental biosynthetic functions.

Regarding compartmental distribution, the cytoplasm and mitochondria contained the highest proportions of robust metabolites relative to their total active metabolites (cytoplasm: 104/517; mitochondria: 26/141) (Fig. 1c, i). In contrast, the endoplasmic reticulum (ER) and ER membrane each harbored five robust metabolites, alongside two and four non-robust compounds, respectively (Fig. 1c, i). The mitochondrial membrane contained only three non-robust metabolites, suggesting its relative resilience under increased production demand (Fig. 1c, i). In terms of the overall robust metabolites, more than 90% are from either cytoplasm or mitochondria; however, the relative percentage of non-robust metabolites compared to robust metabolites is reduced in cytoplasm and increased in mitochondria (Fig. 1c, ii and iii).

We next investigated the reorganization of metabolic fluxes in response to elevated metabolite production demands. For each target metabolite, extensive flux rewiring including metabolite activation, inactivation, and quantitative modulation of flux magnitudes, allowed the network to adapt to increased biosynthetic requirements (Fig. 1d). Relative to the minimal medium control condition, an average of 131 metabolites were newly activated and an equal number were deactivated, while 118 exhibited increased and 168 decreased production flux levels (Fig. 1d). Notably, the number of metabolites with reduced production was significantly greater than in any other category (*t*-test, *P* < 0.05 for all comparisons: activated vs. decreased, deactivated vs. decreased, increased vs. decreased). In contrast, metabolites with elevated flux, indicative of adaptive upregulation, were significantly fewer (*t*-test, *P* = 1.1e-4 for activated vs. increased; *P* = 7.7e-4 for deactivated vs. increased; *P* < 0.05 for decreased vs. increased) (Fig. 1d).

Although numerous metabolites were activated or deactivated under elevated production demands, their associated total flux values remained comparatively low (activated: mean = 0.01037, SD = 0.048 in the overproduced condition; deactivated: mean = 2.8e-4, SD = 0.001 in the control) (Fig. 1e). In contrast, metabolites exhibiting increased or decreased flux displayed substantially higher flux magnitudes with greater variability (increased: mean = 0.048, SD = 0.188; decreased: mean = 0.206, SD = 1.04) (Fig. 1e). Notably, reactions with unchanged fluxes carried the highest overall fluxes (mean = 0.56, SD = 2.29), indicating that core metabolic pathways maintained stable, high-throughput activity despite network-wide perturbation (Fig. 1e).

Metabolic flux rewiring associated with network robustness also led to distinct, context-dependent excretion responses for several metabolites, the majority of which showed activated exchange fluxes (42 out of 48). This includes the secretion of several amino acids and related compounds such as L-phenylalanine, L-cysteine, Ala-Gly, Ala-Leu, and ethyl pyruvate, which were activated across hundreds of robust metabolite conditions. Thymidine and L-alanine secretion were activated in 78 and 59 conditions, respectively (Fig. 1f). However, nearly half of the exchange metabolites were activated in fewer than 10 conditions. In contrast, only two exchange metabolites (Mg²^+^ and chloride) were deactivated in any condition: Mg²^+^ was deactivated in all robust conditions, while chloride[e] was deactivated in 34 conditions. Two metabolites, sodium and proton (H^+^), exhibited increased flux in 5 and 16 conditions, respectively. Meanwhile, sodium, Mn²^+^, and potassium showed decreased flux relative to the control condition in 32, 34, and 34 robust conditions, respectively.

### Metabolic network robustness and response dynamics across the network

**Fig 2:**
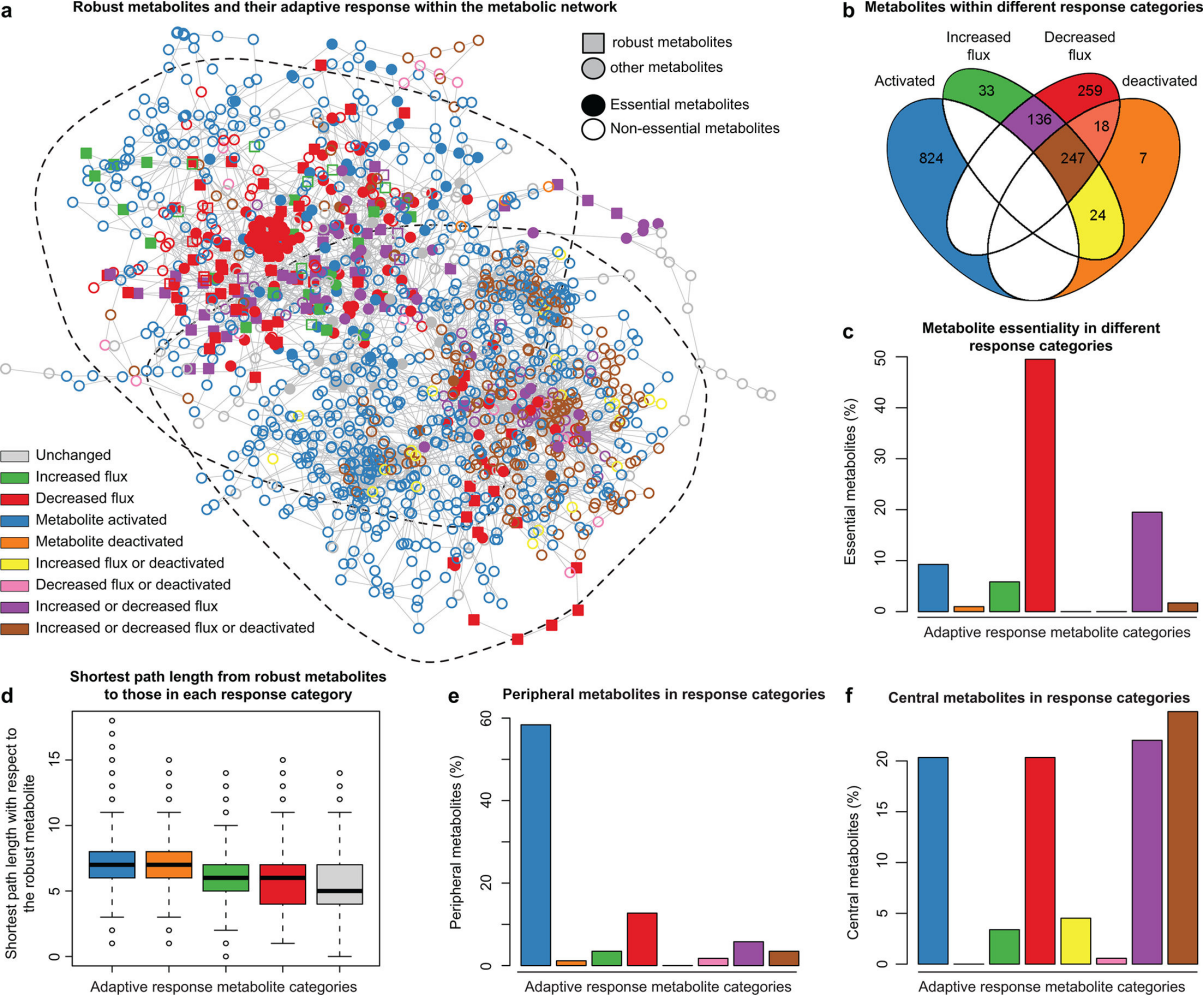
Hierarchical architecture of metabolic network robustness. (**a**) Active metabolic network showing reactions with nonzero fluxes (>10⁻⁴ mmol/gDW/h) under different metabolite overproduction conditions, highlighting robust metabolites and their adaptive responses across central and peripheral pathways. (**b**) Venn diagram illustrating metabolites that are activated, deactivated, or show increased or decreased production fluxes in response to robust metabolite overproduction. (**c**) Proportion of essential metabolites within each response category. (**d**) Shortest path length from robust metabolites to those in each response category within the metabolic network. (**e and f**) Distribution of peripheral (e) and central (f) metabolites across response categories.

Using the Yeast9 metabolic model, we constructed a condensed metabolic network based on active reactions (flux >1 × 10⁻⁴ mmol/gDW/h), wherein metabolites were connected through their associated biochemical reactions (Fig. 2a). Of the 4,130 reactions in the original model, 2,656 carried non-zero flux under at least one simulated condition (flux >1 × 10⁻⁴ mmol/gDW/h) and were retained for network construction. To minimize topological bias, macromolecules (e.g., DNA, RNA, proteins, lipids, carbohydrates), cofactors (H^+^, H₂O, ATP, ADP, AMP, Pi, coenzyme A, CO₂, NADH, NAD^+^), extracellular metabolites, ions, self-connections, duplicate reactions, and highly connected hub metabolites (>50 connections) were excluded. The resulting active network was initially partitioned into 40 topological clusters, of which 21 were small and disconnected from the main component (≤2 metabolites) and subsequently removed. The final network comprised 1,468 metabolites (nodes) and 4,485 reactions (edges), organized into 19 topological modules (Fig. 2a).

We next analyzed network structure and robustness by mapping 144 robust metabolites and their associated perturbed counterparts, annotating each as essential or non-essential to assess links between robustness and cellular viability. Among the 19 topological modules of the metabolic network, the largest contained 369 metabolites, including 27% robust metabolites, which together accounted for 70% of all robust metabolites in the network (101/144) (Fig. 2a). This concentration of robust metabolites within a single, highly connected module suggests that metabolic robustness is a collective property of the core network, rather than a distributed feature across all modules. In contrast, the second-largest module (348 metabolites) contained only two robust metabolites, reflecting functional specialization and peripheralization of non-robust processes (Fig. 2a).

We identified 824 metabolites that were activated in response to the overproduction of at least one robust metabolite (Fig. 2b). These metabolites were distinct from those deactivated or exhibiting flux changes, indicating the presence of latent adaptive subnetworks that are mobilized under elevated biosynthetic demands. In comparison, only 7 metabolites were strictly deactivated, 33 showed increased flux, and 259 exhibited reduced flux (Fig. 2b). Another 247 metabolites demonstrated context-dependent behavior, being deactivated in some conditions but showing altered flux in others, highlighting regulatory flexibility (Fig. 2b). A further 136 metabolites remained active in control conditions but displayed variable fluxes across overproduction demands, suggesting adaptive rerouting that underlies the network’s capacity to maintain metabolic balance (Fig. 2b). Regarding essentiality, nearly half of the metabolites with strictly reduced flux were essential, reflecting their participation in core biosynthetic routes that are indispensable for growth (Fig. 2c). In contrast, those with increased or decreased flux across conditions contained only about 20% essential metabolites, suggesting a role in auxiliary or redundant pathways that provide metabolic flexibility (Fig. 2c). Other metabolite groups exhibited very low essentiality, likely due to their condition-specific functions within the network (Fig. 2c).

To further assess the structural organization of these responses within the metabolic network, we calculated the shortest path lengths between each robust metabolite and all other metabolites. Activated and deactivated metabolites were located significantly farther from robust metabolites (mean and median: activated = 6.82 and 7; deactivated = 7.08 and 7), consistent with their peripheral network localization (Fig. 2d). By contrast, metabolites with increased or decreased fluxes were positioned more centrally (mean and median: increased = 5.76 and 6; decreased = 5.91 and 6), while those with unchanged fluxes were closest to robust metabolites (4 and 5), representing the stable metabolic backbone that persists under perturbation (Fig. 2d). Metabolites with increased or decreased fluxes were significantly closer to robust metabolites than those switched on or off (Wilcoxon rank-sum test, *P* < 0.05; Cohen’s *d* = 0.54), whereas the difference between the activated and deactivated groups was negligible (Cohen’s *d* ≈ 0) (Fig. 2d). Network eccentricity (Fig. 2e) and betweenness analyses (Fig. 2f) further revealed a hierarchical organization of robustness. *Hierarchical architecture is defined as a multilevel structural organization within the network in which distinct features are distributed across different levels, with each level contributing to the overall properties and performance of the system*. Approximately 69% of peripheral metabolites were either strictly activated (43.5%) or displayed reduced flux (25.5%) under overproduction stress (Fig. 2e), suggesting that peripheral reactions function as adaptive buffers that absorb metabolic perturbations. In contrast, central metabolites exhibited diverse responses, including activation (20.3%), flux reduction (20.3%), flux variability (22.0%), and conditional activity or deactivation (24.9%) (Fig. 2f). Most central metabolites remained active under control conditions, reinforcing their role in maintaining basal metabolic fluxes necessary for growth in minimal media (Fig. 2f).

Together, these results reveal that metabolic robustness in yeast emerges from an interplay between structural modularity and functional plasticity. The central metabolic core provides stability and continuity of essential fluxes, whereas peripheral regions confer adaptive flexibility to meet varying metabolic demands. This hierarchical organization enables the yeast metabolic network to maintain growth and productivity despite extensive flux perturbations, illustrating how system-level robustness arises from the coordinated behavior of central and peripheral metabolism.

### Metabolic pathways associated with adaptive response

**Fig 3:**
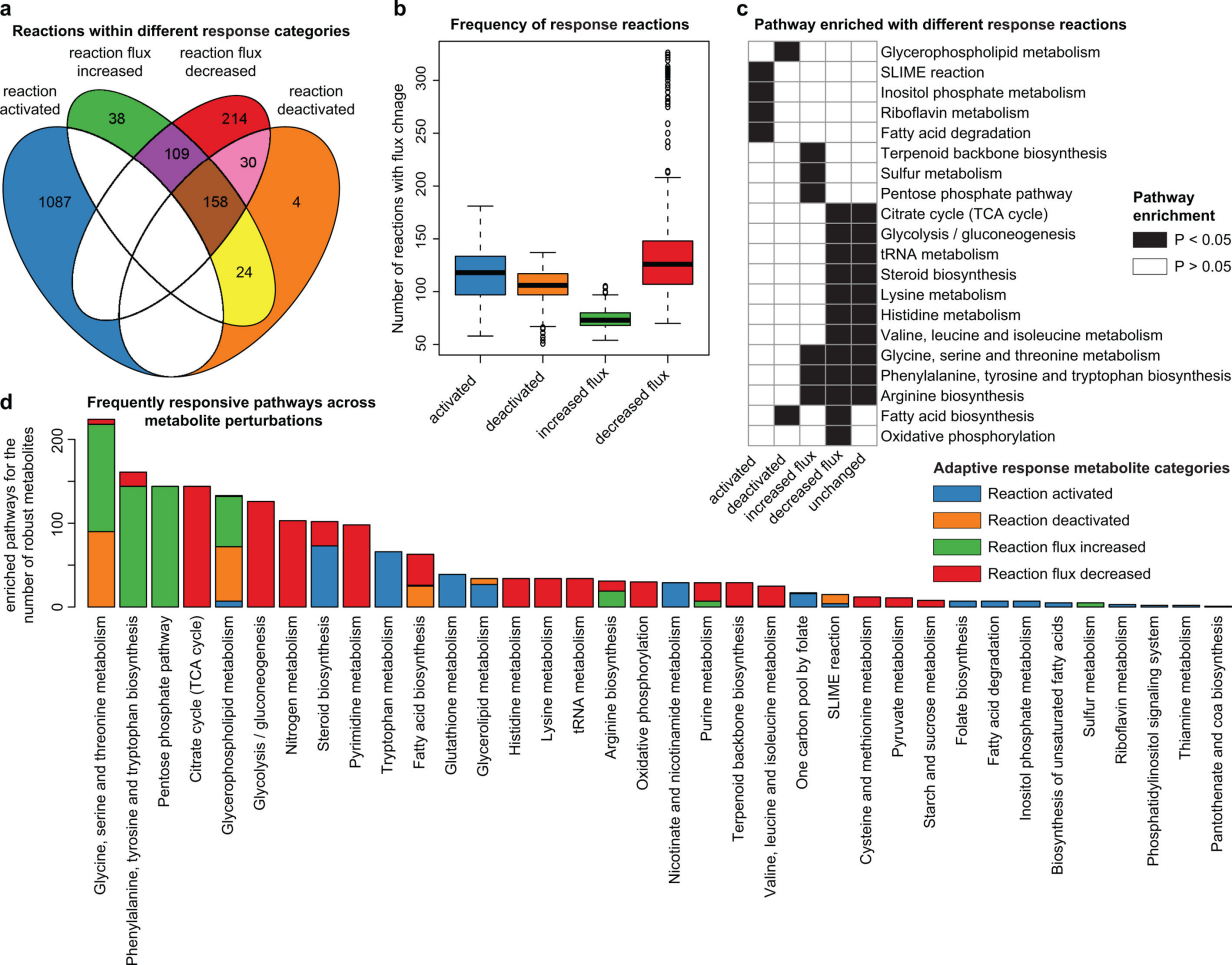
Metabolic pathway association for network robustness and different adaptive responses. (**a**) Venn diagram illustrating the overlap of metabolic reactions among different adaptive response categories, including reactions that are activated, deactivated, or exhibit increased or decreased production fluxes in response to robust metabolite overproduction. (**b**) Boxplot summarizing, for each robust metabolite, the number of reactions from each adaptive response category, highlighting the extent of metabolic reprogramming under elevated metabolite demand. (**c**) Heatmap depicts pathway enrichment for metabolic reactions exhibiting distinct adaptive responses, along with (**d**) the frequency of pathway-level responsiveness across different robust metabolites, indicating the pathways most frequently involved in adaptive metabolic adjustments.

We next examined metabolic pathways underlying adaptive responses associated with metabolic robustness under metabolite overproduction stress. Metabolic reactions that were activated, deactivated, or exhibited increased or decreased flux across simulated overproduction conditions were identified. Consistent with metabolite-level behavior, activated reactions (*n* = 1087) were largely condition-specific, reflecting context-dependent network adaptation, whereas only four reactions were consistently deactivated across all conditions (Fig. 3a). In total, 214 reactions exhibited reduced flux, while 38 showed a consistent increase (Fig. 3a). The remaining reactions displayed context-dependent dynamics, alternating between states depending on the specific overproduction demand (Fig. 3a).

At the level of individual robust metabolites, a substantially larger number of reactions exhibited flux reduction, while a comparable number were newly activated to support alternative biosynthetic routes (Fig. 3b). In contrast, reactions with increased flux were significantly fewer than those that were activated, deactivated, or flux-decreased (Fig. 3a). This pattern indicates that metabolic robustness is primarily achieved through the redistribution and reactivation of alternative reactions rather than amplification of flux through existing pathways. Such a mechanism reflects a conservative adaptive strategy, whereby the yeast metabolic network maintains homeostasis by rerouting metabolic fluxes instead of overburdening individual reactions.

We performed metabolic pathway enrichment analysis to identify functional trends among reactions that were activated, deactivated, or exhibited increased or decreased flux under different overproduction conditions (Fig. 3c). Reactions with decreased fluxes were primarily enriched in central metabolic pathways, including glycolysis/gluconeogenesis, the tricarboxylic acid (TCA) cycle, and multiple amino acid biosynthetic routes (e.g., glycine, serine, and threonine; phenylalanine, tyrosine, and tryptophan; valine, leucine, and isoleucine; histidine; lysine; and arginine metabolism) (Fig. 3c). Pathways related to steroid biosynthesis, tRNA metabolism, fatty acid biosynthesis, and oxidative phosphorylation were also enriched (Fig. 3c). Except for fatty acid biosynthesis and oxidative phosphorylation, most of these pathways also contained reactions with unchanged fluxes, indicating that core metabolism remains largely stable, with only a moderate reduction in flux to balance energy use during overproduction (Fig. 3c). The oxidative phosphorylation pathway, uniquely enriched in decreased-flux reactions, suggests a controlled downregulation of respiratory activity to conserve energy under metabolic stress (Fig. 3c).

In contrast, reactions showing increased fluxes were enriched in six pathways, including terpenoid backbone biosynthesis, sulfur metabolism, and the pentose phosphate pathway, which were uniquely upregulated (Fig. 3c). These reflect a targeted reinforcement of precursor supply and redox balance required to sustain biosynthetic output. Additional pathways—glycine, serine, and threonine metabolism; phenylalanine, tyrosine, and tryptophan biosynthesis; and arginine biosynthesis—exhibited context-specific activation, indicating conditional robustness within amino acid metabolism (Fig. 3c). Reactions that were newly activated were significantly enriched in inositol phosphate metabolism, riboflavin metabolism, fatty acid degradation, and *SLIME reactions*, pointing to an increased need for membrane remodeling, cofactor production, and energy release during adaptive responses (Fig. 3c). Conversely, deactivated reactions were enriched in glycerophospholipid metabolism and fatty acid biosynthesis, reflecting a temporary suppression of lipid anabolism (Fig. 3c). Together, these enrichment patterns reveal a hierarchical metabolic response, in which core energy and biosynthetic pathways are moderated, while peripheral and cofactor-generating routes are selectively activated. This coordinated flux redistribution enables the yeast metabolic network to preserve metabolic stability while enhancing adaptive capacity under overproduction demands.

We examined how frequently each metabolic pathway was re-wired to enhance network robustness by identifying the number of robust metabolites showing significant pathway changes such as activation, inactivation, or major shifts in reaction flux (Fig. 3d). Across most robust metabolites, four key pathways consistently showed increased fluxes: *glycine, serine, and threonine metabolism; phenylalanine, tyrosine, and tryptophan biosynthesis; pentose phosphate pathway*; and *glycerophospholipid metabolism* (Fig. 3d). The *pentose phosphate pathway* displayed exclusively increased fluxes, underscoring its essential role in providing NADPH and ribose-5-phosphate for biosynthesis and redox balance under robust metabolic conditions (Fig. 3d). In contrast, *glycine, serine, and threonine metabolism* and *glycerophospholipid metabolism* exhibited both activated and deactivated reactions, indicating flexible flux rerouting to regulate amino acid interconversion, lipid remodeling, and one-carbon metabolism in response to metabolic stress. Moreover, pathways such as *steroid biosynthesis*, *glutathione metabolism*, *glycerolipid metabolism*, and *nicotinate and nicotinamide metabolism* were frequently activated, suggesting a coordinated enhancement of redox buffering, membrane adaptation, and stress response mechanisms that sustain metabolic stability. These pathways are connected with pentose phosphate pathways that are essential for re-routing fluxes for optimal growth under stress conditions. Conversely, energy- and precursor-generating pathways, including the *citrate cycle (TCA cycle*), *glycolysis/gluconeogenesis*, *nitrogen metabolism*, and *pyrimidine metabolism,* were substantially downregulated across numerous conditions for metabolite overproduction, reflecting a global shift toward reduced energy turnover and biosynthetic demand. Additionally, the consistent suppression of *histidine metabolism*, *lysine metabolism*, *tRNA metabolism*, *oxidative phosphorylation*, *terpenoid backbone biosynthesis*, and *valine, leucine,* and *isoleucine metabolism* further indicates a systemic reallocation of resources from growth-associated processes toward metabolic robustness and stress resilience (Fig. 3d).

### Commonly affected metabolites in most over-produced conditions

**Fig 4:**
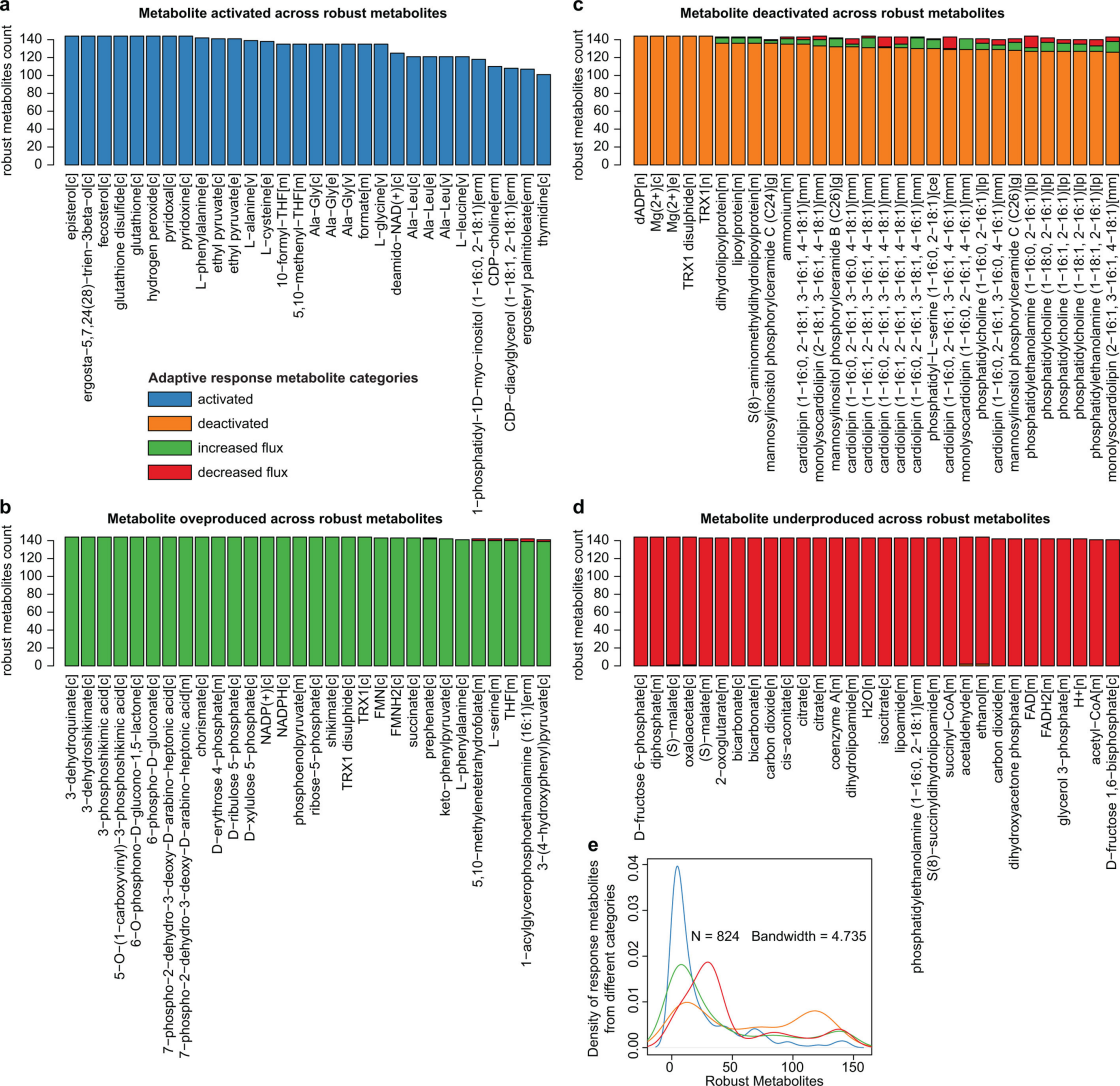
Most frequent adaptive response metabolites. (**a**) Top 30 activated, (**c**) deactivated, (**b**) flux increased, and (**d**) flux decreased metabolites across overproduction demand conditions. (**e**) Density distribution of response metabolites by category among overall robust metabolites.

We subsequently investigated the metabolites modulated through network reprogramming that contribute to enhanced network robustness under diverse overproduction stress conditions. The most consistently activated metabolites were associated with elevated sterol and lipid biosynthesis, reinforced redox buffering through the glutathione system, and augmented cofactor and amino acid metabolism (Fig. 4a). Concurrent activation of folate turnover and membrane remodeling pathways further underscored a shift toward increased biosynthetic flexibility and stress resilience (Fig. 4a). Moreover, metabolites exhibiting elevated flux commonly reflected intensified activity within the pentose phosphate and shikimate pathways, facilitating NADPH regeneration, aromatic amino acid biosynthesis, and redox cycling to sustain anabolic capacity and antioxidant defense (Fig. 4b). In contrast, metabolites commonly deactivated across stress conditions, including cardiolipins, sphingolipids, and lipoyl-proteins, indicated attenuated mitochondrial function, membrane turnover, and other energetically demanding processes, consistent with a transition toward stress-adaptive metabolic states (Fig. 4c). Similarly, decreased production level of intermediates from the tricarboxylic acid (TCA) cycle, glycolysis, and oxidative phosphorylation revealed a global suppression of central carbon metabolism and respiratory activity, reflecting a strategic shift toward energy conservation and redox homeostasis that underpins metabolic robustness (Fig. 4d). These responses are most common under overproduction conditions; however, responsive metabolites across all categories are unevenly distributed across conditions, indicating a non-uniform, condition-dependent pattern (Fig. 4e).

### Metabolic robustness under increasing biosynthetic demand

**Fig 5:**
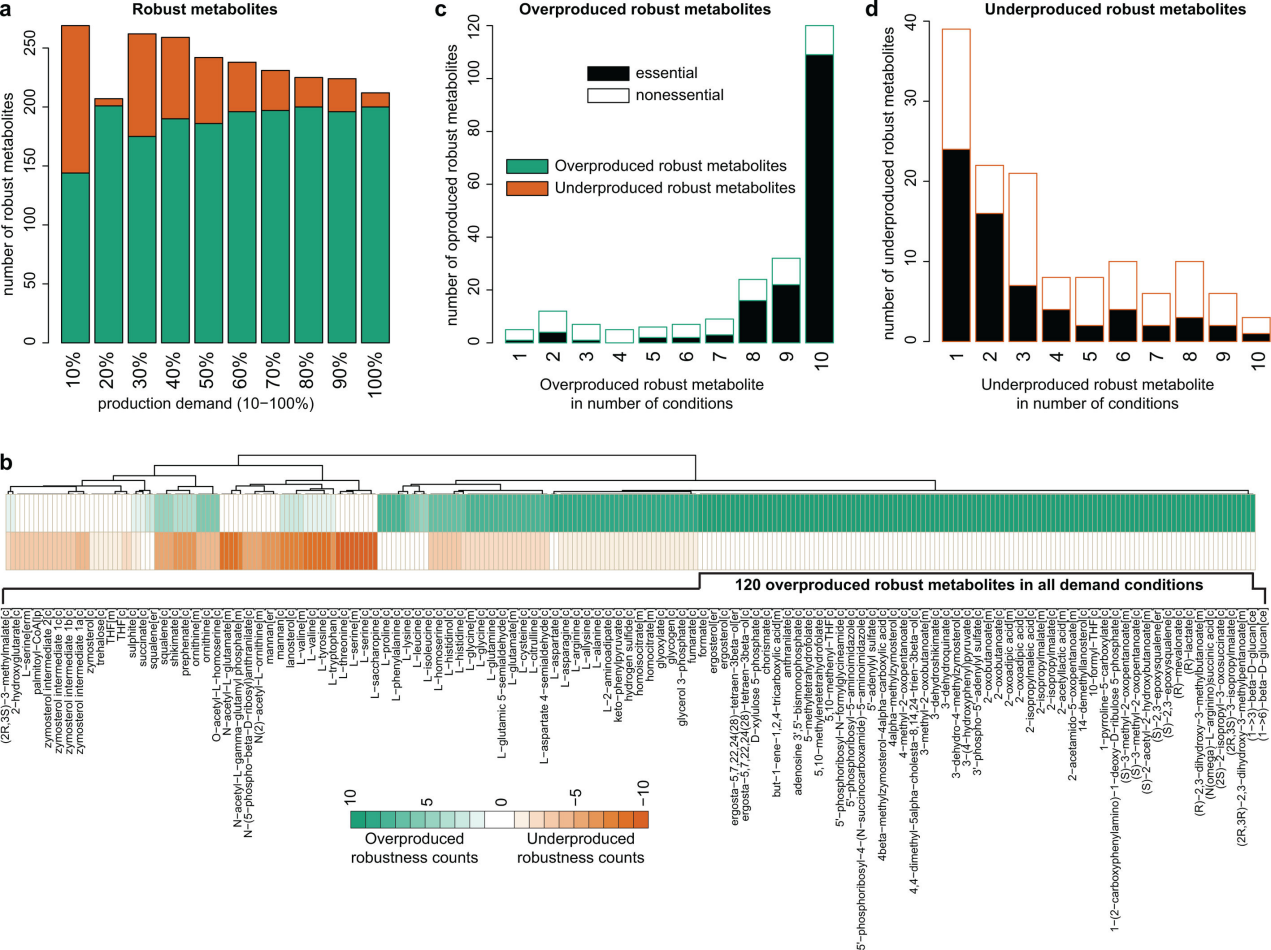
Metabolic network robustness under increasing flux demand. (**a**) Total number of robust metabolites, including both overproduced and underproduced metabolites, across a range of increasing flux demand conditions. (**b**) Frequency of overproduced robust metabolites and (**c**) underproduced robust metabolites across over-demand conditions, highlighting differences in adaptive network responses. (**d**) Heatmap summarizing the global robustness landscape, showing metabolites that are either overproduced or underproduced across all conditions. The 120 core central metabolites are shown, illustrating the metabolic network’s ability to sustain growth while maintaining robustness under all escalating metabolic demands.

We finally examined the extent to which the metabolic network sustains increasing biosynthetic demands for robust metabolites by progressively increasing the flux demand through sink reaction from 10% to 100% of the total production flux, while keeping nutrient availability and growth rate constant. The network maintained robustness across a broad range of metabolites; however, the total number of robust metabolites decreased with increasing demand (Fig. 5a). The number of overproduced robust metabolites, however, increased, indicating a shift toward pathways supporting enhanced biosynthetic flux (Fig. 5a). The metabolic network demonstrated robustness for a substantial number of metabolites (120) under increasing demand conditions (10%–100%) (Fig. 5b), while only three metabolites exhibited the ability to be underproduced without affecting growth (Fig. 5c). Essentiality analysis showed that these 120 global overproduced robust metabolites included the highest fraction (>90%) of essential compounds, whereas underproduced metabolites were comparatively less essential (Fig. 5b and c). Functionally, the universal overproduced robust metabolites were primarily associated with core biosynthetic pathways, including amino acid, nucleotide, lipid, carbohydrate, and sterol biosynthesis, representing the central metabolic network of yeast (Fig. 5d).

## DISCUSSION

One of the defining features of metabolic systems is their intrinsic robustness, a fundamental property of all biological systems (5). This intrinsic robustness enables metabolic networks to adapt and maintain functionality in the face of perturbations (6–9, 24). Metabolic network robustness arises from the structural organization of the network and reflects the inherent redundancy, availability of alternative pathways, and connectivity encoded in the network (6, 7, 18, 20, 23). In metabolic network modeling, including both constraint-based and kinetic frameworks, robustness is typically assessed by introducing perturbations such as altering reaction capacities or completely deleting reactions (18, 24, 28, 32). The effects of these perturbations are evaluated by examining how flux distributions reorganize to maintain a defined cellular objective, such as biomass production. One approach involves altering reaction capacities, for example, by adjusting flux bounds in constraint-based models or varying enzyme kinetic parameters in kinetic models. In this case, reactions are constrained rather than removed, allowing identification of reactions or enzymes that can tolerate reduced or increased capacity while still supporting overall system function. However, attributing robustness to individual metabolites is less straightforward, as metabolites typically participate in multiple reactions. Consequently, perturbations at the reaction or enzyme level do not map uniquely to a single metabolite, making metabolite-specific robustness difficult to define directly. Alternatively, robustness can be assessed through deletions of reactions, genes, or metabolites (11, 34). These knockout analyses classify components as essential or non-essential, where non-essential components either have no impact on growth or can be compensated for by alternative pathways, reflecting inherent network redundancy (11, 34). However, because this approach relies on complete removal rather than graded perturbation, it does not directly reveal which metabolites can accommodate reduced availability or adapt to increasing metabolic demand.

In this study, we present a comprehensive analysis of metabolic network robustness in *S. cerevisiae*. We systematically identify robust metabolites by perturbing metabolite production through the introduction of a hypothetical sink reaction, which is designed to mimic increased metabolite demand or overproduction, and then examine how the network responds to these metabolite-level perturbations. A related approach has been applied in *E. coli*, where metabolite-level robustness was assessed by analyzing fluctuations in metabolite production (flux-sum) in response to the deletion of active, non-essential reactions (34). Conceptually, the sink reaction used in our framework represents the combined effect of modulating one or more reactions associated with a given metabolite. *In vivo*, such changes could occur through altered enzyme expression or activity under conditions of increased metabolite demand. Although this formulation captures a biologically plausible scenario, the fixed production demand imposed for each metabolite in our model is a theoretical assumption and may not be directly achievable under specific environmental or genetic conditions. Furthermore, integrating enzyme kinetic parameters and regulatory constraints into the metabolic model can improve its predictive accuracy by incorporating mechanistic limitations on reaction rates and condition-specific control of metabolic fluxes.

We evaluated the overproduction demands of 317 high-flux intracellular metabolites, excluding macromolecules, cofactors, ions, and highly connected hub metabolites, and found that 85% exhibit robust behavior, underscoring the remarkable resilience of the yeast metabolic network (Fig. 1b). Among the robust metabolites, over half (144/269) increased flux production to meet elevated demands, with most of these compounds being essential for growth (Fig. 1), indicating that robustness is an intrinsic feature of the metabolic core (7, 9, 19, 25). This intrinsic robustness highlights an evolved design principle in which core metabolic functions are stabilized through distributed redundancy and dynamic reconfiguration of flux pathways (21). The remaining robust metabolites (125/269) reduced their production to accommodate metabolite waste, thereby maintaining growth. We systematically assessed the reprogramming of the metabolic network in response to 144 overproduced robust metabolites.

Robust metabolites, predominantly localized in the cytoplasm and mitochondria, were located within a specific, largest module of the metabolic network (Fig. 1c and 2a). Their concentration within a single topological module suggests that structural redundancy and extensive cross-linking enable compensatory flux rerouting when local reactions are perturbed (19). This finding is consistent with theoretical predictions from network biology, in which resilience arises from modular organization and overlapping reaction routes that buffer perturbations and prevent systemic collapse (20, 35, 36). Topological analysis of the metabolic network revealed a hierarchical organization of robustness, highlighting distinct adaptive responses across the system (Fig. 2). Under perturbation, a distinct set of metabolites became activated that were inactive under control conditions (Fig. 2b), and these newly activated metabolites occupied more distant, peripheral positions relative to the perturbed robust metabolite (Fig. 2d). Overall, nearly half of the peripheral metabolites were activated under conditions of high metabolic demand, functioning as adaptive buffers that absorb fluctuations and prevent disturbances from propagating into the core network (Fig. 2e). These peripheral subnetworks thus function as dynamic shock absorbers, maintaining overall network stability (20, 24, 37). In contrast, metabolites exhibiting moderate increases or decreases in flux under perturbation were positioned closer to the robust metabolites, occupying more central regions of the network (Fig. 2a, d, and f). Notably, nearly half of the essential metabolites reduced their fluxes to accommodate network robustness (Fig. 2c). Together, these patterns suggest a clear division of labor between stability and adaptability: central metabolism ensures continuity and robustness of essential functions, while peripheral modules provide flexibility and enable dynamic adjustment to environmental or metabolic stress.

Flux-based analyses revealed that metabolic robustness in yeast is primarily achieved through large-scale reorganization of reaction activities rather than uniform amplification of existing fluxes (Fig. 3a). Under perturbed conditions, the network activates alternative routes while downregulating energetically costly pathways, thereby redistributing fluxes to maintain system balance (Fig. 3). The predominance of decreased or newly activated fluxes reflects a conservative adaptive strategy in which metabolic stability is maintained through flux redistribution rather than increased production (18, 38) (Fig. 3a and b). The emergence of newly active reactions further reveals latent adaptive circuits that expand the network’s functional capacity under stress. These context-dependent pathways expand the metabolic solution space and serve as reserve circuits that can be mobilized to buffer metabolic stress, underscoring the latent plasticity of the yeast metabolic network (23, 39). This pattern also suggests that most reactions under control conditions already operate near their optimal capacity (23, 25, 40, 41), making further flux increases unfeasible; thus, activating alternative metabolites or reactions becomes a more effective strategy for the metabolic system to adapt under stress.

A hallmark of robust adaptation was the coordinated suppression of central energy metabolism, including glycolysis, the tricarboxylic acid (TCA) cycle, and oxidative phosphorylation, accompanied by a targeted reinforcement of redox and cofactor-generating pathways (Fig. 3c, d, and 4d). This global shift suggests that yeast adopts a metabolic strategy favoring energy conservation and redox stability over maximal throughput under stress (21). Concurrently, the consistent upregulation of the pentose phosphate and shikimate pathways across diverse conditions underscores the central role of NADPH generation in sustaining redox homeostasis and anabolic potential (Fig. 3d and 4b) (21). NADPH serves as a universal reducing equivalent that fuels lipid, nucleotide, and amino acid biosynthesis while maintaining antioxidant capacity (42, 43). Upregulation of these pathways suggests that redox balance represents a key organizing principle of metabolic robustness, ensuring that the network remains functionally stable even when energy-intensive reactions are downregulated (Fig. 3c and d).

The recurrent activation of sterol, lipid, folate, and glutathione metabolism (Fig. 4a) further highlights the coupling of membrane maintenance, cofactor regeneration, and redox buffering as integral components of the adaptive response. Enhanced sterol and phospholipid biosynthesis likely contributes to membrane fluidity and stability during flux reorganization, while elevated folate and glutathione turnover support one-carbon metabolism and redox detoxification (Fig. 4a). These processes collectively sustain the structural and redox integrity of the cell under metabolic perturbation. Additionally, the frequent secretion of amino acids and organic acids under most of the robust conditions (Fig. 1f) suggests that metabolite excretion functions as an overflow mechanism to relieve intracellular accumulation and rebalance redox potential (44). Such export-mediated buffering is consistent with observed overflow metabolism in microbial systems, where excess intermediates are excreted to stabilize internal homeostasis (44–46). Together, these responses illustrate how metabolic robustness encompasses both intracellular flux redistribution and extracellular metabolite management to preserve system stability.

We further probed the limits of metabolic network robustness by progressively increasing the flux demand for each robust metabolite from 10% to 100% (Fig. 5). The network remained resilient to elevated demand for over a hundred central metabolites, primarily associated with core biosynthetic pathways, including amino acid, nucleotide, lipid, carbohydrate, and sterol biosynthesis (Fig. 5). Notably, more than 90% of these universally robust metabolites are essential for growth, underscoring their fundamental role in sustaining cellular function. In contrast, only a small subset of metabolites was consistently underproduced across all demand conditions, with most underproduced metabolites displaying context-specific responses (Fig. 5).

Collectively, our findings indicate that metabolic robustness in yeast arises from three interdependent principles: stabilization of a central core network that anchors essential functions, activation of peripheral subnetworks that enable adaptive rerouting, and energetic and redox optimization that sustains biosynthetic capacity while limiting stress-induced damage. Together, these features form a distributed control strategy that preserves growth and redox homeostasis across diverse perturbations, highlighting robustness as an emergent property of coordinated network dynamics rather than a single protective mechanism. Overall, this work establishes a quantitative and mechanistic framework for understanding metabolic robustness as a systems-level phenomenon, showing that yeast achieves resilience through coordinated flux reprogramming, redistribution of metabolic control, and selective modulation of energy and redox balance. This hierarchical architecture allows the network to maintain essential biosynthetic fluxes and cellular homeostasis under substantial perturbation. The principles identified here, including core stability, peripheral flexibility, and energetic optimization, define a unifying systems paradigm for robust metabolism, offering conceptual and practical insights into how biological networks maintain function under stress and how such robustness can be leveraged in metabolic engineering.

## SUPPLEMENTAL MATERIAL

File S1 msystems.00276-26-s0001.csv Metabolites evaluated for robustness.

ASM does not own the copyrights to Supplemental Material that may be linked to, or accessed through, an article. The authors have granted ASM a non-exclusive, world-wide license to publish the Supplemental Material files. Please contact the corresponding author directly for reuse.
